# Wastewater modeling of Cr (VI) adsorption by sulfonated lignin of pine cones: equilibrium, kinetics, and isotherm study

**DOI:** 10.1186/s13065-026-01763-8

**Published:** 2026-03-17

**Authors:** Suhad A. Yasin

**Affiliations:** Chemistry Department, College of Science, University of Duhok, Duhok, Kurdistan Region 42001 Iraq

**Keywords:** Adsorption, Hexavalent chromium removal, Bio-adsorbent, Sulfonated lignin

## Abstract

Water is vital to life, so preserving it from pollution is an important issue. Chromium, in its various oxidation states, poses a significant threat to water contamination. This study analyzes the sulfonation process, which improves the Cr (VI) adsorption capability of lignin (a bio-adsorbent generated from pine cones), to produce sulfonated lignin (SL). The optimal pH for Cr (VI) adsorption was 2. Within the first ten minutes, uptake was rapid; however, it slowed thereafter and eventually reached equilibrium after 30 min. The relationship between the initial and equilibrium concentrations of Cr (VI) was linear. The percentage of Cr (VI) removal increased as the SL dosage increased. The adsorption on SL was better explained by the Lagergren pseudo-second-order kinetic model than by the Elovich or intraparticle diffusion models. The adsorption data were better correlated by the linear form of the Langmuir-2 isotherm model than by the Freundlich and Tempkin models. The maximum monolayer sorption capacity *(Qm)* from the Langmuir isotherm model was determined to be 34.714 mg/g. The high monolayer capacity of SL implies that it is a highly effective adsorbent for Cr (VI). SL is a cost-effective, eco-friendly, and promising adsorbent with outstanding potential for removing Cr(VI) contaminants from polluted water.

**Graphical abstract:**

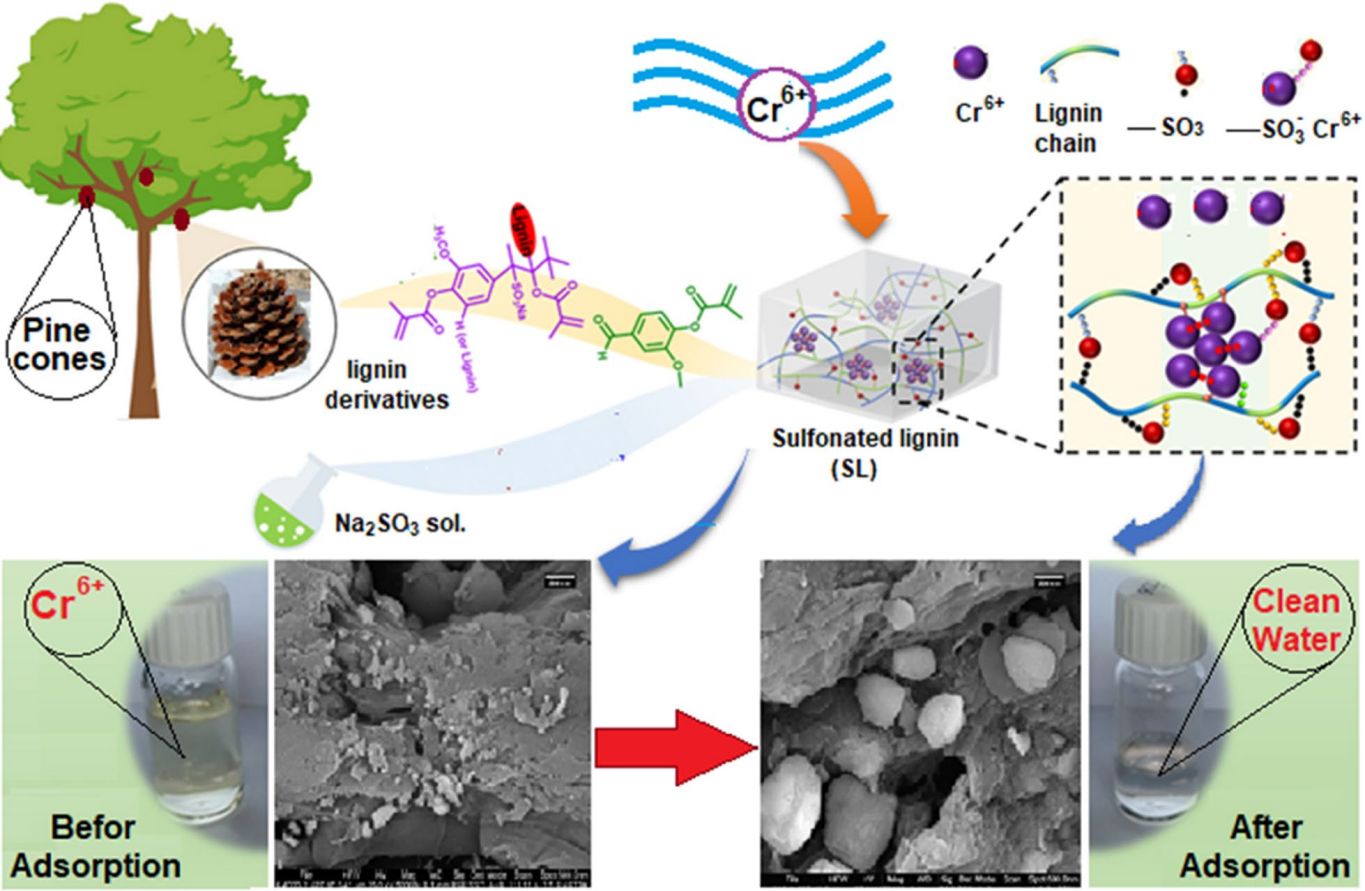

## Introduction

There are many types of pollution that may affect different parts of our environment, but the vital concern is human health, and consequently, it is at greater risk due to pollution [1, 2]. Of the many contaminants that are jeopardizing our environment, one is hexavalent chromium, which not only imposes toxicology and health effects on the contaminated but also affects the economic impact and technical feasibility of regulating it more stringently [3]. One example of the impact of pollution on the economy is the regulation imposed by the California Office of Environmental Health Hazard Assessment, which has set a target of 0.02 ppb or less of chromium (VI) in water as a public health goal. This level is unlikely to be detected using current water analysis and purification technologies [4, 5]. The Environmental Protection Agency of the United States and the World Health Organization have recommended a limit for Cr concentration in natural waters of below 50 mg L⁻¹ in drinking water [6, 7]. Nonetheless, the range of chromium concentrations in natural waterways is rather wide, ranging from 5.2 to 208,000 mg L^− 1^ [8].

Chromium (VI), which is recognized as the third most common component at hazardous waste sites, has both toxic and carcinogenic hazards. Consequently, remediation measures are frequently required to mitigate the risks it presents to human health and the environment [9, 10].

Accordingly, hexavalent Cr (VI) and trivalent Cr (III) are the only forms of Cr that are harmful to humans [11]. The reduction-oxidation equation in which Cr (VI) can be converted to Cr (III) and vice versa is [12]:

$${\mathrm{C}}{{\mathrm{r}}_2}{\mathrm{O}}_{7}^{2}+{\text{ 14}}{{\mathrm{H}}^+}+{\text{ 6 electrons}}~ \to {\text{2Cr }}\left( {{\mathrm{III}}} \right){\text{ }}+{\text{ 7}}{{\mathrm{H}}_2}{\text{O }}+{\text{ 1}}.{\text{33 eV}}$$

The high oxidizing power of Cr (VI) and the poor oxidizing power of Cr (III) are manifested in the above equation. As can be observed from the equation, the Cr (III) requires 1.33 eV to be oxidized to Cr (VI), which is considered extremely high inside the living system and hence unavailable. Hence, the forward reaction is spontaneous, while the reverse reaction cannot happen inside the human body [13]. Chromium, regardless of its oxidation state, is non-biodegradable, and its release into streams and lakes results in bioaccumulation in living creatures, posing health risks to animals, plants, and humans [14].

Despite the fact that chromium can exist in a variety of oxidation states, the prevalent species are (III) and (VI). They are interconvertible; for example, the naturally occurring chromium state is (III), which gets oxidized to the (VI) state in the industry, the latter state pollutes the environment in this state mainly [15]. When humans are subjected to Cr (VI), it penetrates into the blood and is rapidly taken up by erythrocytes and reduced to Cr (III) by glutathione inside the red blood cells [16]. Cr (VI) is enough to make the red blood cell membrane permeable to it. While the larger-sized Cr (III) gets trapped inside the blood cells, leading to the formation of reactive intermediates by complexing to DNA [17].

There have been several conventional methods for wastewater treatment to eliminate heavy metal contamination. These methods are chemical precipitation [18], chemical coagulation [19], ion exchange [20, 21], activated carbon adsorption [22–24], solvent extraction [25], electrodialysis [26], ultrafiltration [27], reverse osmosis [28], separation by a membrane [29–31] and biological treatment [32]. However, these methods are considered expensive [33]. Hence, the search for a low-cost method is a challenging problem. Agricultural waste materials and bio-adsorbents are suitable as low-cost and sustainable materials [34, 35]. Among the heavy metals studied by Febrianto et al. [24] were Cr (III) and Cr (VI), and they employed over twenty different biosorbents; however, only one study made use of Pinus sylvestris. The reviewed studies were conducted between 1999 and 2008 [36].

Miretzky and Cirelli published a comprehensive review in which they surveyed articles that used raw and modified lignocellulosic materials as bio-adsorbents for Cr (VI) and Cr (III) removal. Of the many articles, only a few have used pine or modified pine as bio-adsorbents [37].

Structural modifications of bio-adsorbents are necessary to increase their adsorption capacity by introducing phenolic, carboxyl, or amino functional groups [38]. In a study by Sjostrom [39], sulfonation in acidic sulfite pulping could generate hydrophilic sulfonic groups as well as free phenolic groups. Hokkanen et al. [28] conducted a review of various articles that explored the use of bio-modified cellulose-based adsorbents to enhance adsorption capacity. The authors demonstrated that sulfonation of the lignocellulosic bio-adsorbent effectively improved metal ion adsorption capacity, attributing this enhancement to alterations in the surface functional groups of the bio-adsorbent caused by sulfonation. The enhancement in adsorption capacity can be ascribed to two factors.

Firstly, the increase in the internal and external surfaces by swelling the material and secondly, altering the surface of the biosorbent to enable the entry of polyfunctional organic compounds into the bio-adsorbent matrix in order to enhance the number of binding sites [29–31]. The necessity to use structurally modified bio-adsorbent also evolves from the fact that using functionally modified bio-adsorbent decreases the chemical oxygen demand (COD), a test that shows the extent of pollution of organic materials [32].

Pine cones are considered a sustainable agriculture byproduct with no food value apart from being used for decorative purposes or burned in the stove in winter [40]. The three phenyl-propanol molecules in lignin—p-coumaryl, coniferyl, and sinapyl—are polymerized into a three-dimensional network. Pine cones are categorized as softwoods, distinguished by a lignin level generally exceeding that of hardwoods and agricultural byproducts. Furthermore, softwood lignin typically possesses a greater content of guaiacol units, which exhibit stronger reactivity than syringyl units [34].

The choice of bio-adsorbent depends on two factors, namely low cost and sustainability. Lignin is regarded as the second most abundant natural polymer, following cellulose [41].

This study employs sulfonated lignin derived from pine cones as a bio-adsorbent for the removal of Cr(VI) from aqueous solutions. Adsorption isotherm and kinetic models are developed to elucidate the adsorption behavior and mechanism. The most suitable model is identified to provide insight into the efficiency and mechanism of Cr(VI) adsorption onto sulfonated lignin. Considering that actual polluted water often comprises numerous competing metal ions that could influence adsorption efficacy, it is recommended to examine the ion selectivity of sulfonated lignin in forthcoming research.

## Materials and methods

### Metal solution

Analytical grade chemicals were utilized in this work (Fluka, Germany). Dissolving 1.4144 g of K₂Cr₂O₇ in 1 L of deionized water yielded a (500 mg.L^− 1^) metal stock solution for experimental solution synthesis.

### The preparation of adsorbent

Pine cones were randomly collected from Zaweta village in Duhok city (available unused and considered waste material). The pine cone was pulverized and sieved (500 micrometers) to obtain particles of uniform size. Sulfonation of lignin of pine cone chips was carried out under an acidic condition, as follows: 12.6 g of Na₂SO₃ was dissolved in 200 ml of deionized water and added to 10 g of a pine cone. The pH of the solution was adjusted to 3 through adding (1.0 M) HNO₃. The solution was subsequently agitated at 70 °C for 24 h. Upon completion of the reaction, sulfonated lignin derived from pine cones (SL) was filtered, meticulously washed with deionized water, and subsequently dried overnight at 70 °C in an oven [42].

### Chemical analysis

The concentration of metal ions in wastewater was analyzed using an Atomic Absorption Spectrophotometer (AAnalyst 200, Perkin Elmer). Standard solutions for AAS calibration were prepared from a certified 500 mgL^−1^Cr(VI) stock solution (analytical grade, Sigma-Aldrich) via serial dilution with deionized water to obtain working standards in the range of 1–10 mg.L^− 1^. Calibration was performed prior to each measurement to ensure analytical accuracy and reliability. Each sample was analyzed in triplicate, and the standard deviation of the measurements was ± 1%.

### The test of color-forming reaction

SL was reacted with a colour-forming reagent to detect coniferaldehyde functional groups via a colour-forming reaction [42]. 50 ml of 0.5 g phloroglucinol-dihydride in 95% ethanol and 25 ml of concentrated hydrochloric acid were combined to create the reagent. The sample immediately turned reddish violet when the reagent was introduced dropwise.

### Adsorbent characterization

The adsorbent’s functional groups were examined using Fourier Transform Infrared Spectroscopy (FTIR) within the range of 4000–400 cm⁻¹. The material was combined with potassium bromide (KBr), pulverized into a fine powder, and formed into pellets prior to analysis. The acquired spectra were utilized to discern the distinctive peaks associated with various functional groups on the adsorbent surface.

The surface shape and texture of the adsorbent were analyzed using Scanning Electron Microscopy (SEM). The samples were affixed to aluminum stubs with carbon tape and subsequently coated with a small layer of gold to improve conductivity prior to imaging. SEM micrographs were acquired at different magnifications to examine particle size, surface roughness, and porosity, which are essential characteristics affecting adsorption performance.

### The equilibrium of the adsorption study

Batch experiments were used to investigate the equilibrium adsorption behavior of hexavalent chromium (Cr (VI)) onto sulfonated lignin (SL). In a thermostatically controlled water bath shaker running at 150 rpm for 120 min, a fixed quantity of SL (0.1 g) was added to the Cr (VI) solution and kept at 25 °C. The AAS was used to quantify residual chromium in the solution after adsorption.

### Study of the adsorption isotherm

Different SL adsorbent doses (1, 2, 4, and 8 g.L^− 1^) and the concentrations of Cr (VI) (10, 20, 30, and 40 mg.L^− 1^) were used in adsorption isotherm. Adsorbent doses were prepared by shaking for 120 min at pH 2 and 25 °C.

The equation $\:{q}_{e}$ was employed to quantify the quantities of Cr (VI) that were adsorbed onto the adsorbent (g) [31, 43]:

1 $$\:{q}_{e}=\frac{{(C}_{o}-{C}_{e\:\:})\:\:\:\:V}{M}$$

In this context, $\:{C}_{o}$ and $\:{C}_{e}$denote the initial and equilibrium concentrations of Cr (VI) in mg.L^− 1^, respectively. *V* signifies the volume of the experimental solution, while $\:M$ represents the mass of the SL in grams. The percentage removal of Cr (VI), or adsorption efficiency (R%), is determined by the subsequent equation [38]:

2 $$\:R\%=\frac{{C}_{o}-{C}_{e}}{{C}_{o}}\times\:100$$

## Results and discussion

Modification of the pine cone can be achieved by sulfonation of the enal and enone functional groups of the coniferaldehyde end group of lignin. To check for the existence of a coniferaldehyde group in a pine cone, we have carried out the Wiesner reaction. A reddish-violet hue appeared instantly upon adding the Weiser reagent dropwise to the pine cone, which vanished within 30 min.

**Fig. 1:**
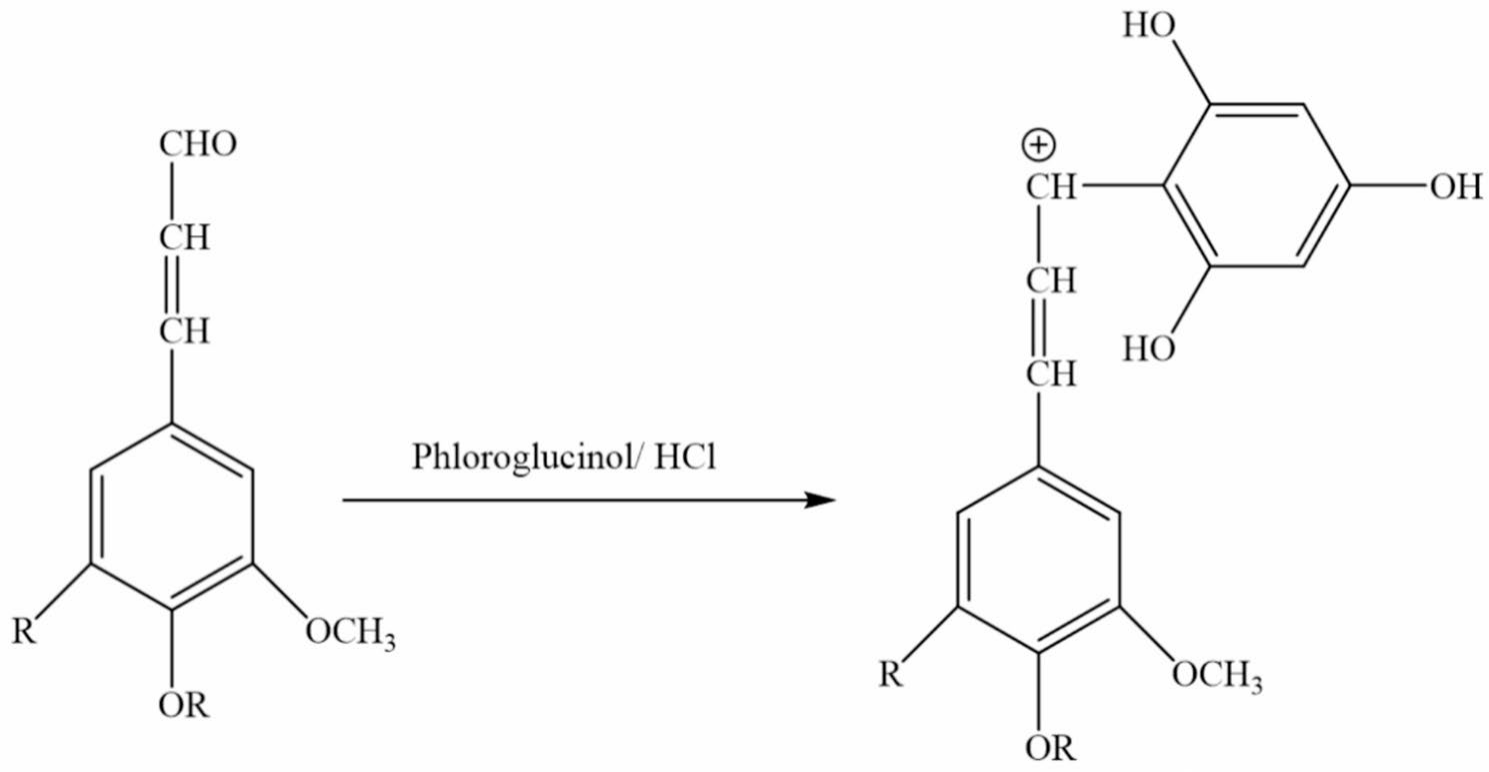
The Weisner reaction

**Fig. 2:**
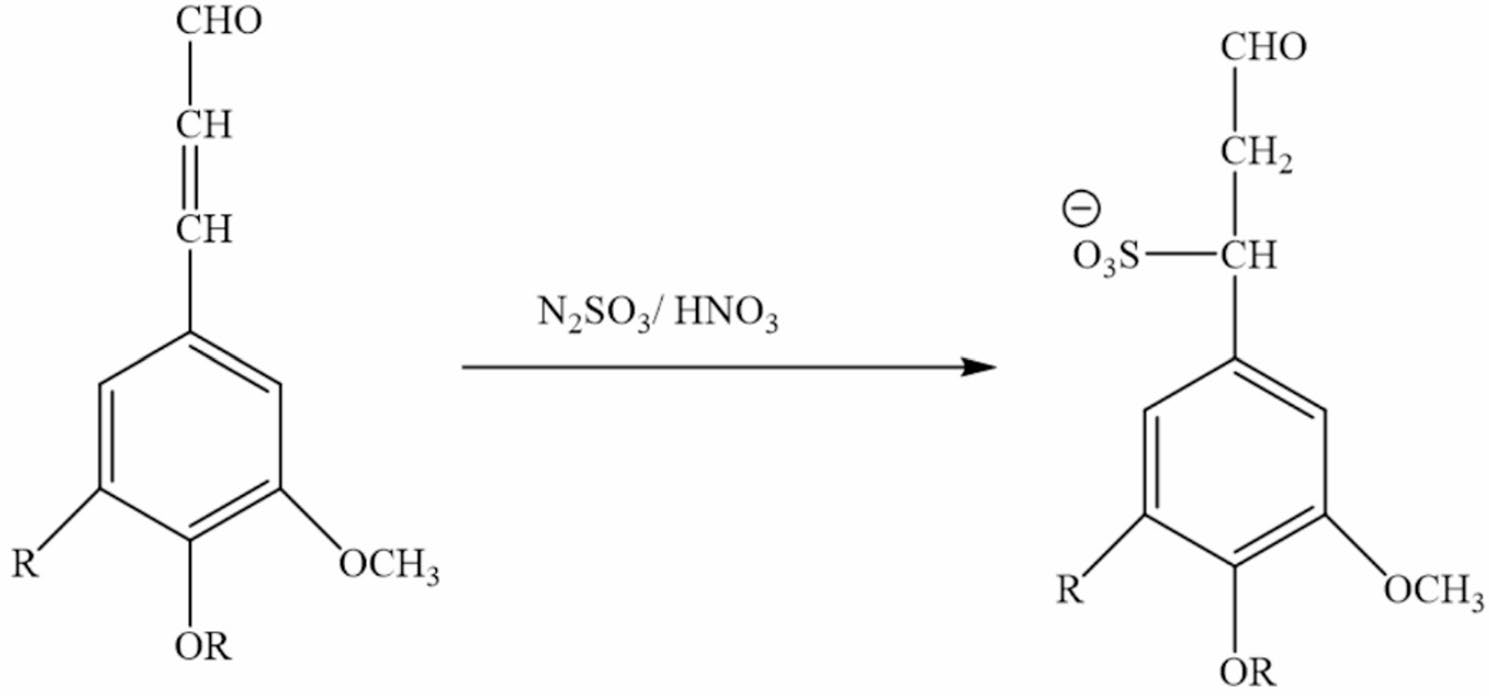
Sulfonation reaction of lignin

**Fig. 3:**
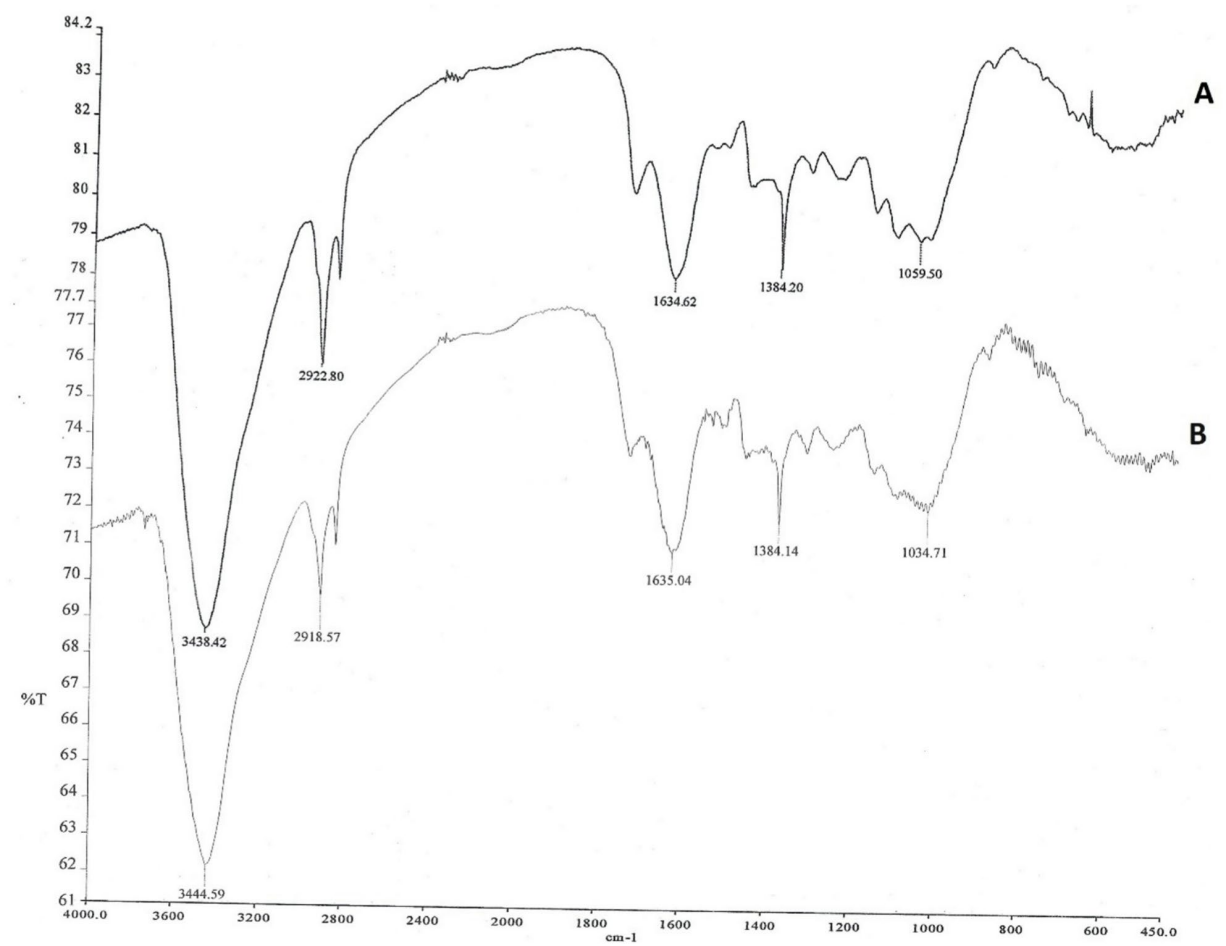
**A** FT-IR spectrum of un-sulfonated lignin (**B**) sulfonated lignin (SL)

The color change of the pine cones confirmed the presence of the coniferaldehyde end group. The suggested equation for the Weisner test reaction is shown in Fig. 1 [44, 45]:

Sulfonation of lignin was carried out by using sodium sulfite. The sulfite ion reacts with the double bond of the enal and enone groups. Figure 2 illustrates the.

equation of the sulfonation step. The Brunauer–Emmett–Teller (BET) surface area analysis study revealed that the surface area of SL is negligible, suggesting that macropores are the primary component, contributing marginally to the overall surface area in comparison to meso- and micropores. Thus, the chemistry of surface functional groups (e.g., -OH and -COOH) has a greater influence on the adsorption of metal ions by SL than physical surface area, with chemical interactions such as chelation and ion exchange serving as the primary mechanisms. Figure 3 gives the FT-IR spectra of unsulfonated lignin and sulfonated lignin, respectively. In this study, the most substantial vibration peak is identified at 3300 cm^− 1^ which denotes the presence of the –OH functional group [46]. Figure 3A shows different band shapes in the region 1000–1630 cm⁻¹. The bands at 1125 and 1037 cm⁻¹ of Fig. 3B are related to the out-of-phase and in-phase stretching of (SO_₃_- H₃O⁺ hydrate), respectively. These bands shape the difference, and the appearance of stretching of the (SO_3_-H_3_O^+^ hydrate) confirms the sulfonation of lignin pine cone [47].

**Fig. 4:**
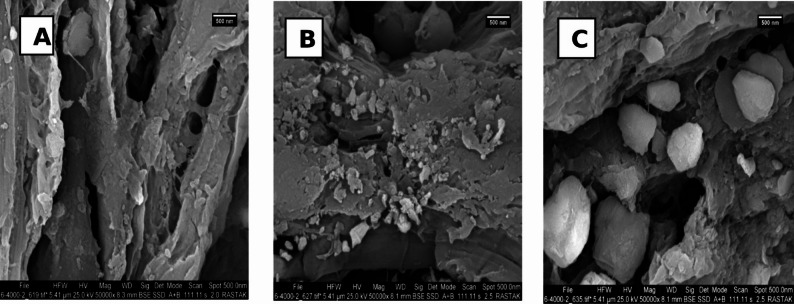
**A** SEM of un-sulfonated lignin; **B** sulfonated lignin SL before adsorption; **C** sulfonated lignin SL after adsorption

The scanning electron microscopy (SEM) analysis findings for pine cones and sulfonated lignin (SL) before and after Cr (VI) adsorption are illustrated in Fig. 4. As depicted in Fig. 4A, the pine cone surface exhibits a rough texture with abundant grooves, channels, and large pores, which are favorable for Cr (VI) adsorption. Figure 4B and C show that following sulfonation and subsequent adsorption, the material’s surface morphology remains largely intact, with the pores and grooves still visible.

### Influence of initial solution pH

**Fig. 5:**
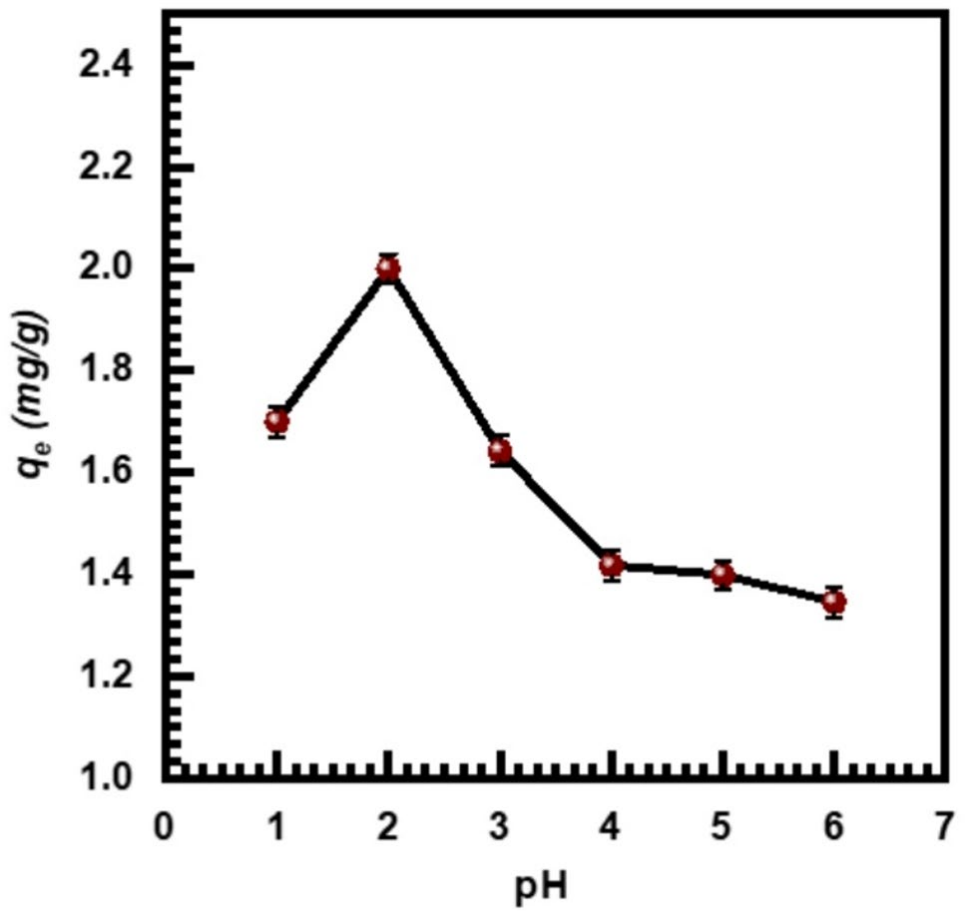
The impact of pH on Cr (VI) adsorption onto SL adsorbent

It is widely recognized that the system pH regulates the adsorption capacity by affecting the surface characteristics of the adsorbent and the ionic metal ions [48]. Consequently, adsorption experiments were conducted across a pH range of 1 to 6, maintaining other parameters constant. Figure 5 illustrates the adsorption of Cr (VI) ions onto the adsorbent as a function of pH.

The highest Cr(VI) adsorption capacity was recorded at pH 2. A gradual decrease in adsorption efficiency was observed with increasing pH, which is consistent with findings reported in earlier studies [49–52]. The type and ionization state of the functional groups on the adsorbent surface, along with the speciation of Cr (VI) in the aqueous phase, are responsible for the pH’s impact on Cr (VI) adsorption. Different types of chromium species coexist between pH values of 1 and 6, such as Cr₂O₇⁻², HCrO⁻¹, Cr₃O₁₀⁻², and Cr₄O₁₃⁻², of which HCrO⁻⁴ predominates [53]. However, it has been documented that H₂CrO₄ can form in conditions that are sufficiently concentrated and acidic. As the solution pH increases, CrO_4_^2^ and Cr_2_O_7_^−2^ become prominent. It has been suggested that a complicated mechanism is involved in the binding of hexavalent chromium, for that reason, we observed the maximum removal of chromium ion (40.0%) occurring at pH (2). In an acidic solution, the surface of the SL becomes strongly protonated, or positively charged which favors the adsorption of Cr (VI) as HCrO_4_^−^ in the anionic form. The net positive surface potential of the adsorbent decreased as a result of the surface becoming less protonated due to the rise in pH. This decreases the electrostatic forces between adsorbent and adsorbate, leading to a reduction in the capacity of adsorption [54].

### The impact of shaking time

50 mL of 10 mg.L⁻¹ Cr (VI) solution and 0.1 g of SL were used to study the influence of shaking time (0–120 min) on Cr (VI) ion adsorption at pH 2 and 150 rpm.

**Fig. 6:**
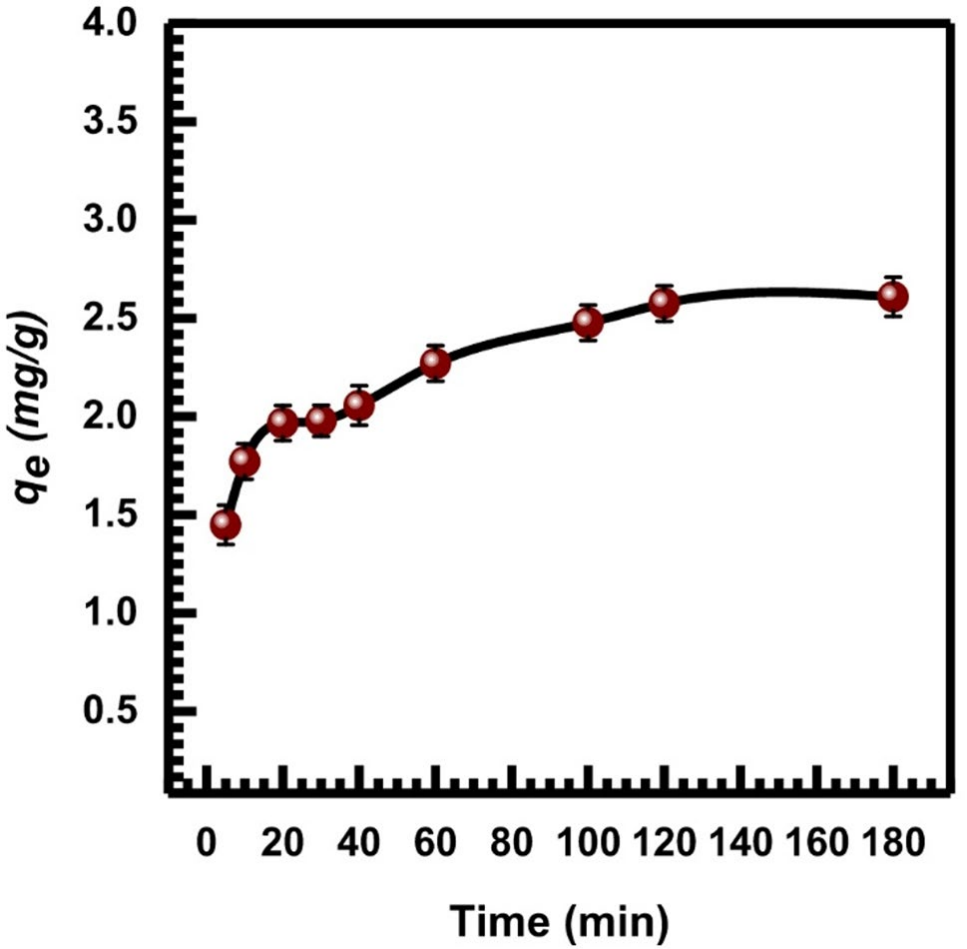
The impact of time on Cr (VI) adsorption onto SL adsorbent

Figure 6 shows that Cr (VI) adsorption was fast in the first ten minutes, but then it slowed down and reached equilibrium after thirty minutes, and then there was no discernible rise in the percentage of adsorption. This might be because the adsorption and desorption processes take place after the adsorbent surfaces are saturated with Cr (VI).

### Effect of initial concentration of Cr (VI)

**Fig. 7:**
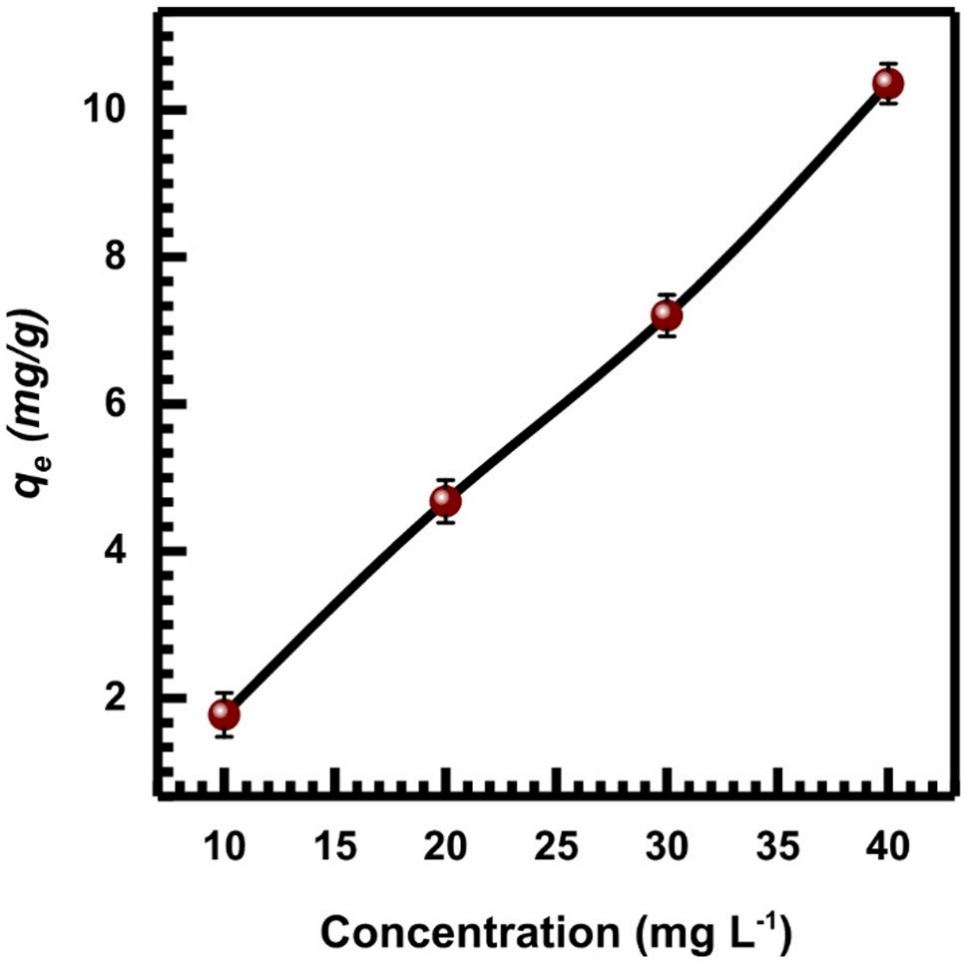
The impact of concentration on Cr (VI) adsorption onto SL adsorbent

The impact of initial Cr (VI) ion concentration was examined utilizing concentrations of (10–40 mg. L^− 1^) of Cr (VI) with a dosage of 2 g.L^− 1^ of SL. The value of *q*_*e*_ grows in relation to the initial concentration of Cr (VI), as seen in Fig. 7.

This is explained by the fact that the initial concentration rises, increasing the mass gradient between the solution and SL. This action drives the movement of Cr (VI) from the bulk solution to the surface of the SL [55, 56]. Due to a rise in Cr(VI) ions for the fixed number of SL adsorbent sites, equilibrium may be reached at higher concentrations [57].

### Effect of SL adsorbent dosage

**Fig. 8:**
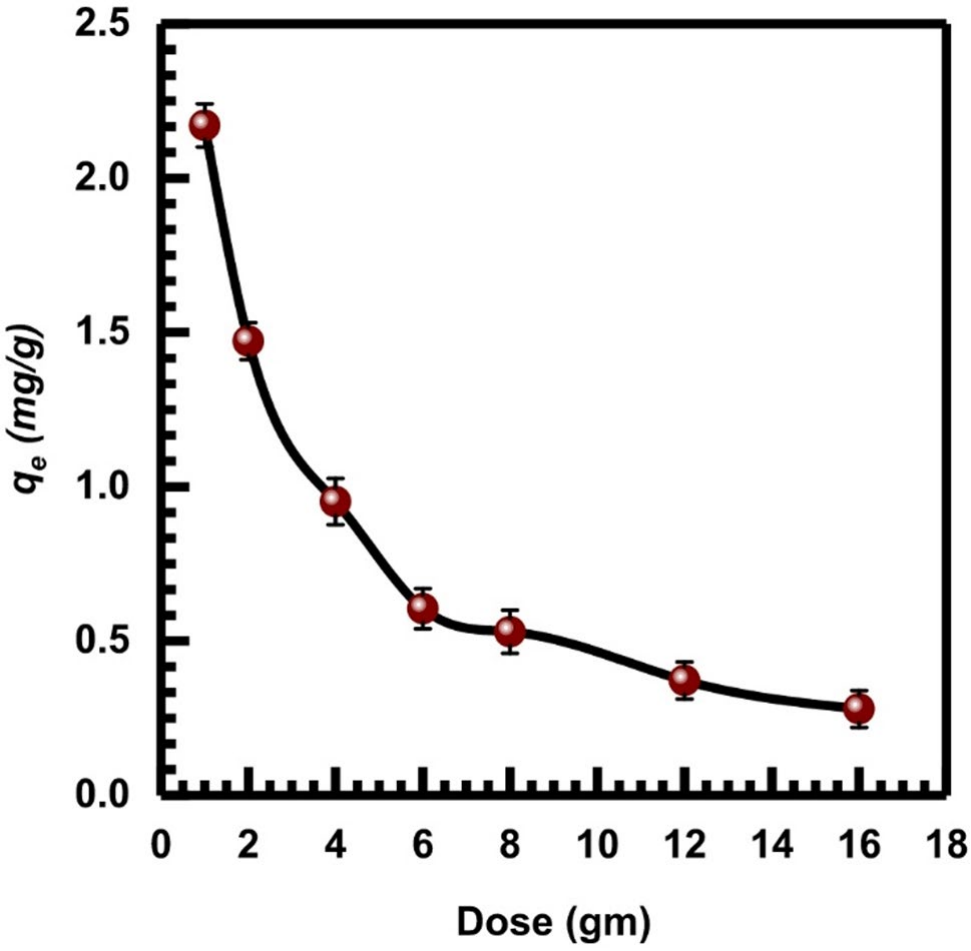
The impact of dose on Cr (VI) adsorption onto SL adsorbent

The impact of adsorbent dosage on the adsorbed amount of Cr (VI) of the concentration of (10 mg.L⁻¹) at room temperature was investigated by using the SL concentration range (1, 2, 4, 6, 8, 12, 16 g.L^− 1^). The elimination percentage of Cr (VI) grows in direct proportion to the dosage of SL, as shown in Fig. 8. The reason behind this is the increase of bonding sites of SL as the dosage increases and the increase of the available surface area of SL.

### Adsorption kinetics

In order to ascertain the residence time necessary for the adsorption reaction to be completed, the heavy metal ion adsorption rate can be analyzed using kinetic data.

Also, kinetics adsorption helps in assessing the dynamics of the adsorption process in terms of the rate constant and the order of reaction; this provides important information for designing and modelling of the adsorption operation [58]. It is the system circumstances and the interaction between the sorbate and the sorbent that determine the adsorption mechanism. Some of the mechanisms involved in adsorption include ion exchange, chelation, physical sorption, and chemical sorption. Several kinetic models have been developed to rationalise the adsorption process of the heavy metal ion on adsorbent surfaces [59].

A simple pseudo-first-order equation due to Lagergren was used by Ho and Mckay [60, 61] with the following form:

3 $$\:\mathrm{log}({q}_{e}-{q}_{t})=\mathrm{log}{q}_{e}-\left(\frac{{k}_{1}}{2.303}\right)t$$

**Fig. 9:**
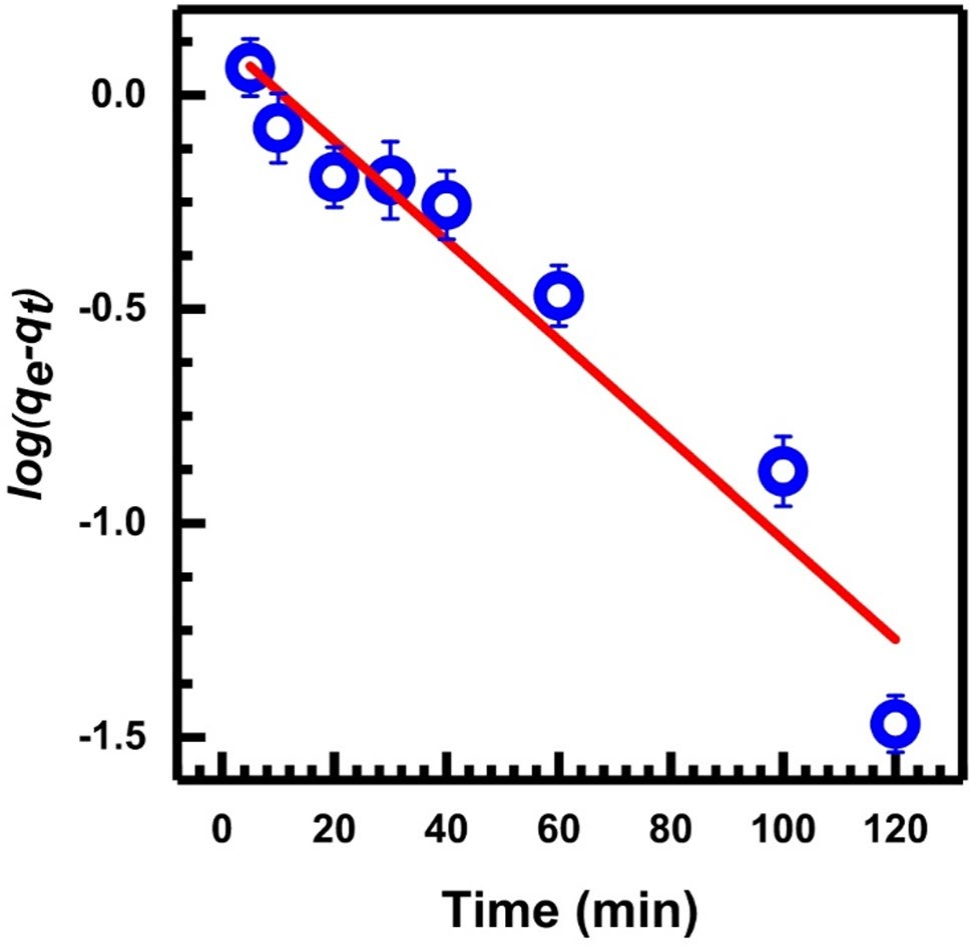
Pseudo-first-order kinetic, Eq. (3)

*q*_*t*_ is the adsorption capacity of adsorbed Cr (mg.L⁻¹) on SL at equilibrium ($\:{q}_{e}$ ) and at any time *t*, respectively, and *k*_*1*_ is the rate constant of pseudo-first-order adsorption (min^− 1^). Figure 9 shows the plot of log (*q*_*e*_-*q*_*t*_) versus time as given by Eq. 3.

Table 1 gives the slope, intercept, correlation coefficient, R², and other calculated parameters from Eq. 3. The kinetic data for Cr (VI) adsorption onto SL is not well characterized by the pseudo-first-order kinetic model, as indicated by the low value of R² 0.9456. Consequently, it becomes necessary to evaluate the applicability of the pseudo-second-order rate Eqs. [55, 56], which has to be tested. The pseudo-second-order equation [60] is linearly expressed:

4 $$\:\frac{t}{{q}_{t}}=\frac{1}{{k}_{2\:}\:{q}_{e}^{2}}+\frac{1}{{q}_{e}}\left(t\right)$$

In this case, the pseudo-second-order adsorption rate constant is *k*_*2*_ (g/mg. min). The following formula can be used to determine the initial adsorption rate, *h* (mg/g. min) [62, 63].

5 $$ h\, = \,k_{{\mathrm{2}}} q_{e} ^{{\mathrm{2}}} $$

**Fig. 10:**
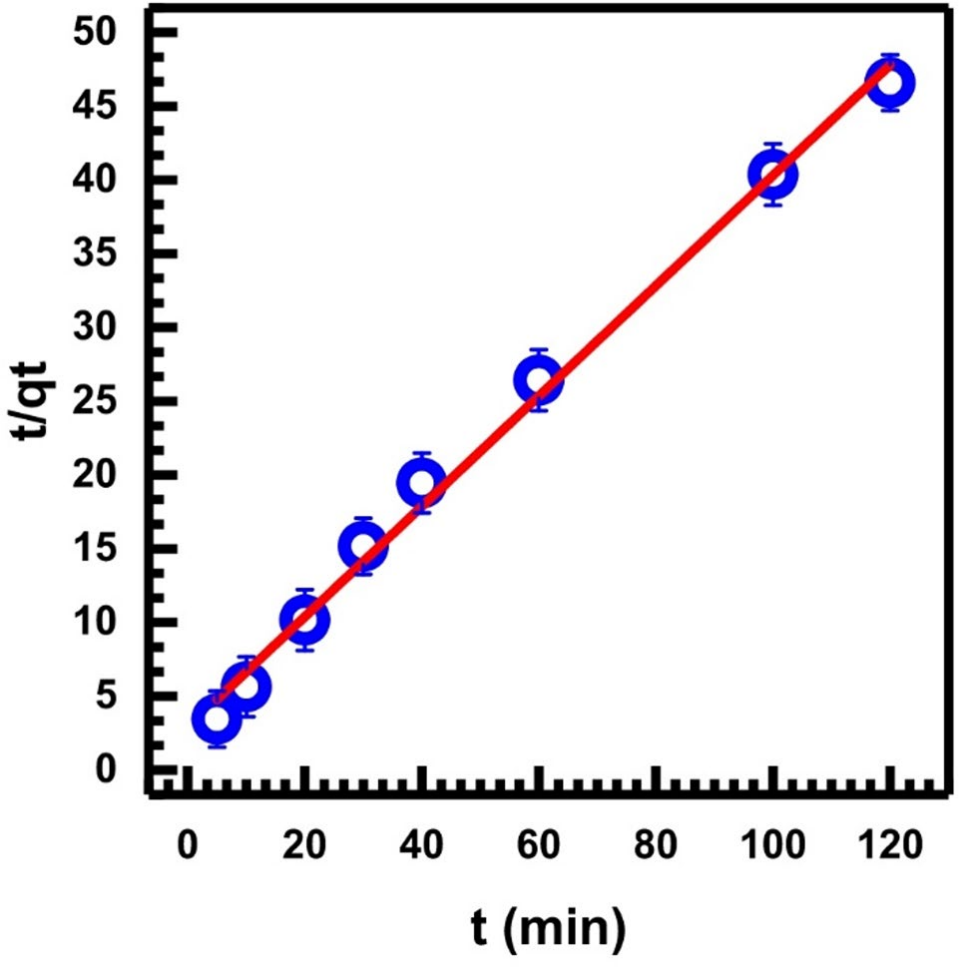
Pseudo-second-order kinetic, Eq. (4)

Figure 10 shows the parameters (t/*q*_*t*_) versus time during the adsorption process as shown in the pseudo-second-order Eq. (4), and Table 1 gives the statistical parameters and the calculated parameters of Eq. 4. The correlation coefficient was found to be (0.994). This high correlation coefficient suggests the Cr(VI) sorption is governed by pseudo-second-order rather than pseudo-first-order kinetics. Hence, we can suggest that the Cr (VI) adsorption onto SL involves a rate-limiting step, which may be chemical adsorption or chemisorption involving valence forces through sharing or exchange of electrons between Cr (VI) and SL as an adsorbent, and provides the best correlation data [61].

In the linear version of the Elovich equation, the Elovich model, which has been modified by Chien and Clayton, can be stated as follows [43, 64]:

6 $$\:{q}_{t}=\frac{1}{\beta\:}\mathrm{ln}(\alpha\:\beta\:)+\frac{1}{\beta\:}\mathrm{ln}\left(t\right)$$

**Fig. 11:**
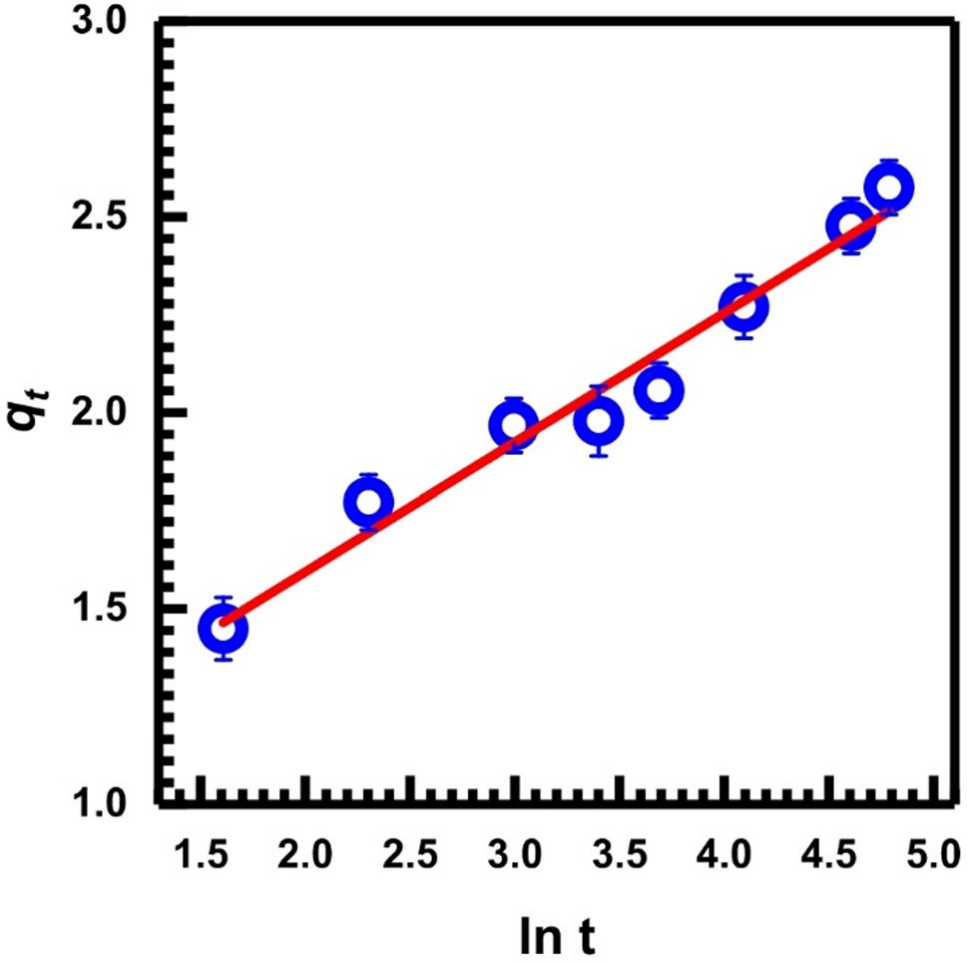
Elovich kinetic, Eq. (6)

This equation represents the initial adsorption rate, denoted by *q*_*t*_, and the desorption constant, denoted by β. Figure 11 illustrates that a linear relationship is produced when the plot of *q*_*t*_ vs. ln(t) is examined. Table 1 presents the statistical parameters and the calculated Elovich constants *α* and β according to Eq. (6).

When studying the rate-determining phase for Cr (VI) adsorption onto SL in a batch process with rapid stirring, it is worth testing the intra-particle diffusion model as a kinetic model.

According to the intra-particle diffusion model, metal ions are transported from the surface of solid particles to their internal pores. There is a high probability that this will be a slow procedure, and it might be the step that determines the rate [65]. Weber and Morris [66] derived the rate of intra-particle diffusion by the relationship between *q*_*t*_ and the square root of time, *t*^1*/*2^, as shown in Eq. (7):

7 $$\:{q}_{t}={K}_{dif}{t}^{\frac{1}{2}}+{B}_{L}$$

**Fig. 12:**
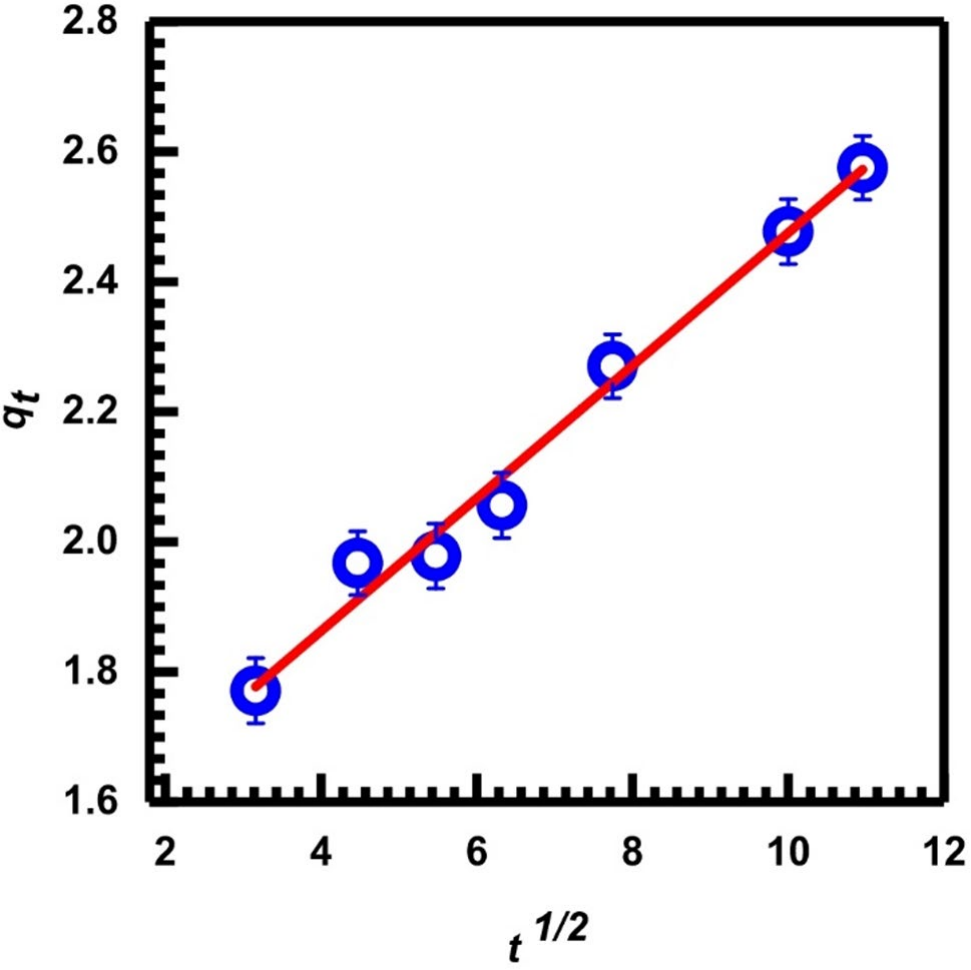
Intra-particle diffusion adsorption, Eq. (7)

*B*_*L*_ is related to the thickness of the boundary layer, and *K*_*dif*_ (mg/ g. min^1*/*2^) is the intra-particle diffusion rate constant. The slope and intercept of the *q*_*t*_ vs. t^1*/*2^ plot are used to derive the *K*_*dif*_ and *B*_*L*_ values, respectively. Table 1 displays the *K*_*dif*_ and *B*_*L*_ values. The adsorption process would entail intra-particle diffusion if the plot of *q*_*t*_ versus *t*^1*/*2^ in Fig. 12 revealed a linear relationship, and it would be the controlling step if this line passed through the origin [67].

**Table 1:** Statistical parameters, calculated (*q*_*e*_ calc.) and experimental (*q* exp) values for kinetics models of (10 mg.L⁻¹ Cr (VI) adsorption onto (2 g.L^−1^SL) at constant temperature

Kinetic model	Parameters	Parameter value
Pseudo-first-order kinetic model (Eq. 3)	Slope	-0.0116
	Intercept	0.1255
	*q* exp (mg/g)	2.609
	*k*_*1*_(min^− 1^)	0.027
	*q*_*e*_ calc.(mg/g)	1.462
	R^2^	0.9456
Pseudo-second-order kinetic model (Eq. 4)	Slope	0.3741
	Intercept	2.91
	*k*_2_ (g/mg. min)	20.807
	*h* (mg/g. min )	148.772
	*q*_*e*_ calc.(mg/g)	2.674
	R^2^	0.9949
Elovich model (Eq. 6)	Slope	0.3301
	Intercept	0.9337
	*q*_*e*_ calc.(mg/g)	3.030
	α (mg/g. min)	6.856
	R^2^	0.9708
Intra-particle diffusion model (Eq. 7)	Slope (K_diff_ (mg/g. min^1*/*2^)	0.1161
	Intercept (B_*L*_(mg/g)	1.3372
	R^2^	0.9565

### Study of adsorption isotherm

A metal adsorbate concentration at equilibrium between the liquid and adsorbed phases at an adsorbent surface is studied in an adsorption isotherm [68].

The adsorption isotherm study is considered to be the primary requirement for designing any adsorption system. Also, it provides valuable information to understand the mechanism of adsorption of chromium ions onto SL at equilibrium. Several isotherm models have been developed; however, in this study we will investigate the applicability of some of these models to model chromium ion adsorption onto SL.

The Langmuir model, which is considered a theoretical model, was first established to study the adsorption of gases onto solids [69]. This model provides invaluable information on the mechanism of adsorption. The Langmuir model assumes complete monolayer coverage on the homogeneous adsorbent surface. Also, the model assumes uniform energies of adsorption onto the surface and no transmigration of the adsorbate [70].

The non-linear Eq. (8) representing the Langmuir adsorption isotherm model is given below [63]:

8 $$\:{q}_{e}=\frac{{Q}_{m}{K}_{l}{C}_{e}}{1+\:{K}_{L}{C}_{e}}$$

Equation (8) can be linearized in four forms as given below.

Langmuir-1:

9 $$\:\frac{{c}_{e}}{{q}_{e}}=\frac{1}{{K}_{L}{Q}_{o}}+\frac{{c}_{e}}{{Q}_{o}}$$

Langmuir-2:

10 $$\:\frac{1}{{q}_{e}}=\frac{1}{{K}_{L}{Q}_{o}}\times\:\frac{1}{{C}_{e}}+\frac{1}{{Q}_{o}}$$

Langmuir-3:

11 $$\:{q}_{e}={Q}_{o}-\frac{{q}_{e}}{{c}_{e}}+\left(\frac{1}{k}\right)$$

Langmuir-4:

12 $$\:\frac{{q}_{e}}{{C}_{e}}=K{Q}_{o}-k{q}_{e}$$

**Fig. 13:**
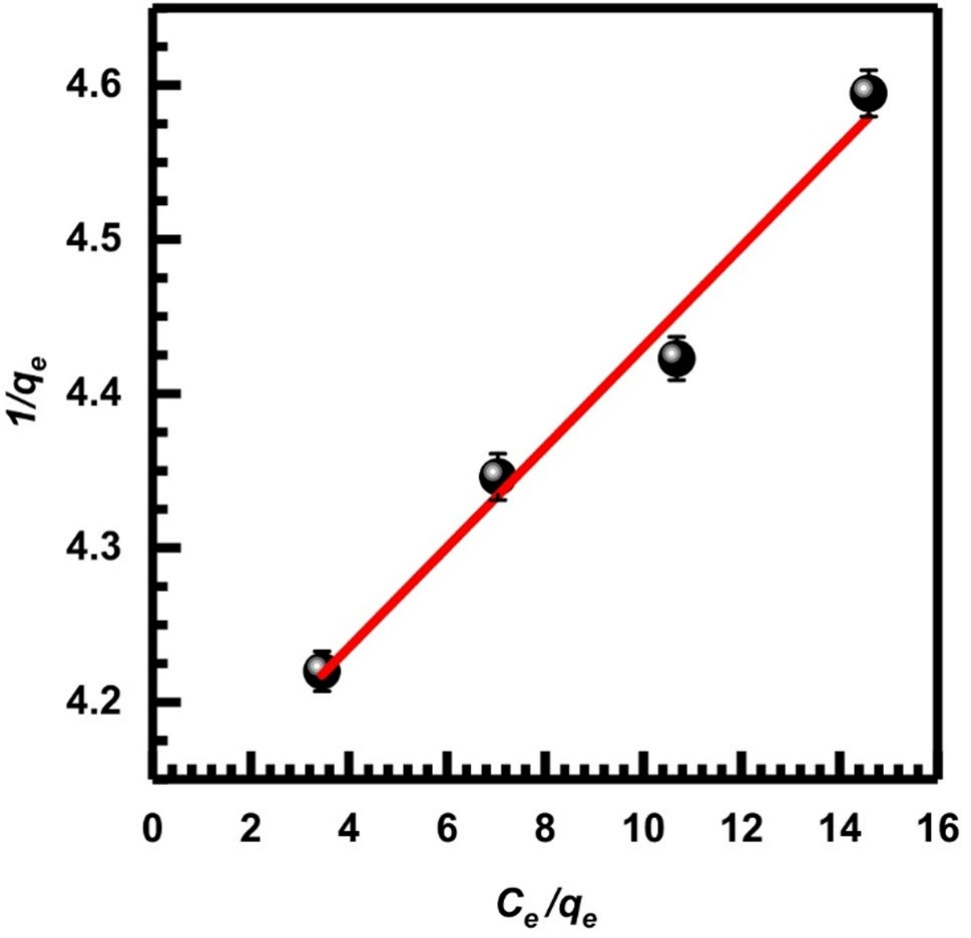
Isotherm of Langmuir-1, Eq. (9)

**Fig. 14:**
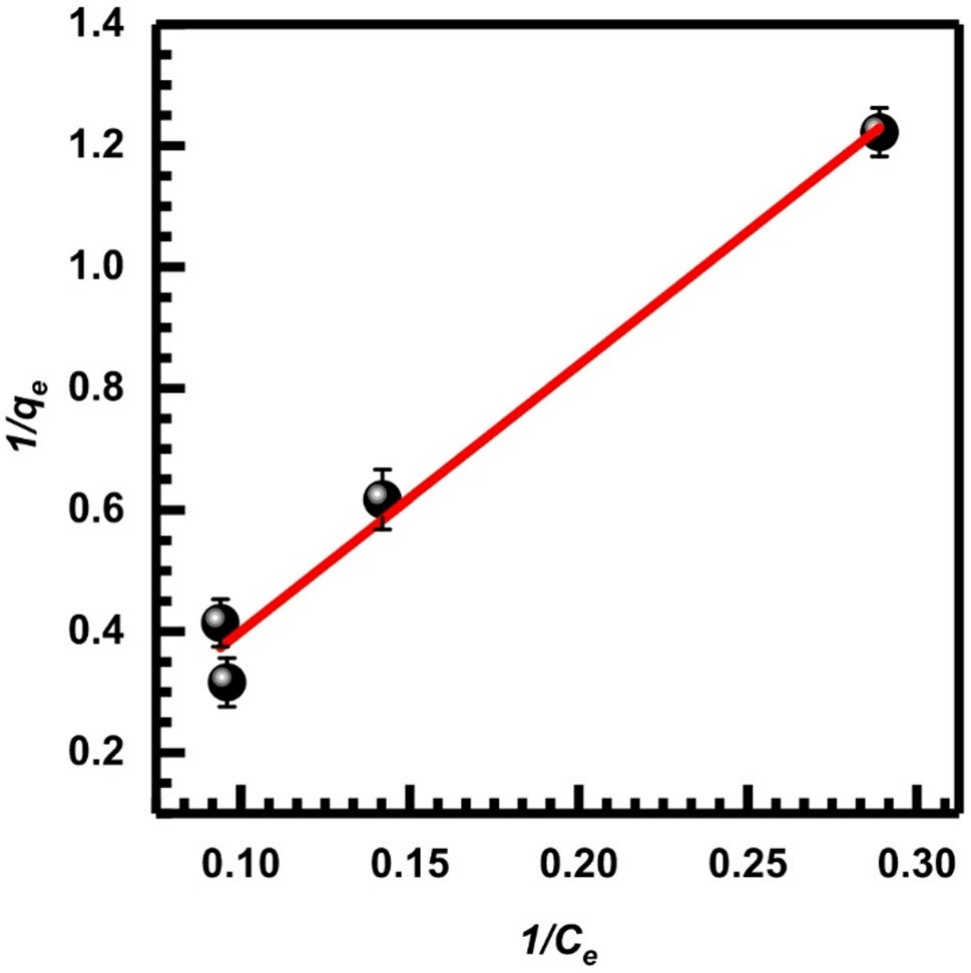
Isotherm of Langmuir-2, Eq. (10)

**Fig. 15:**
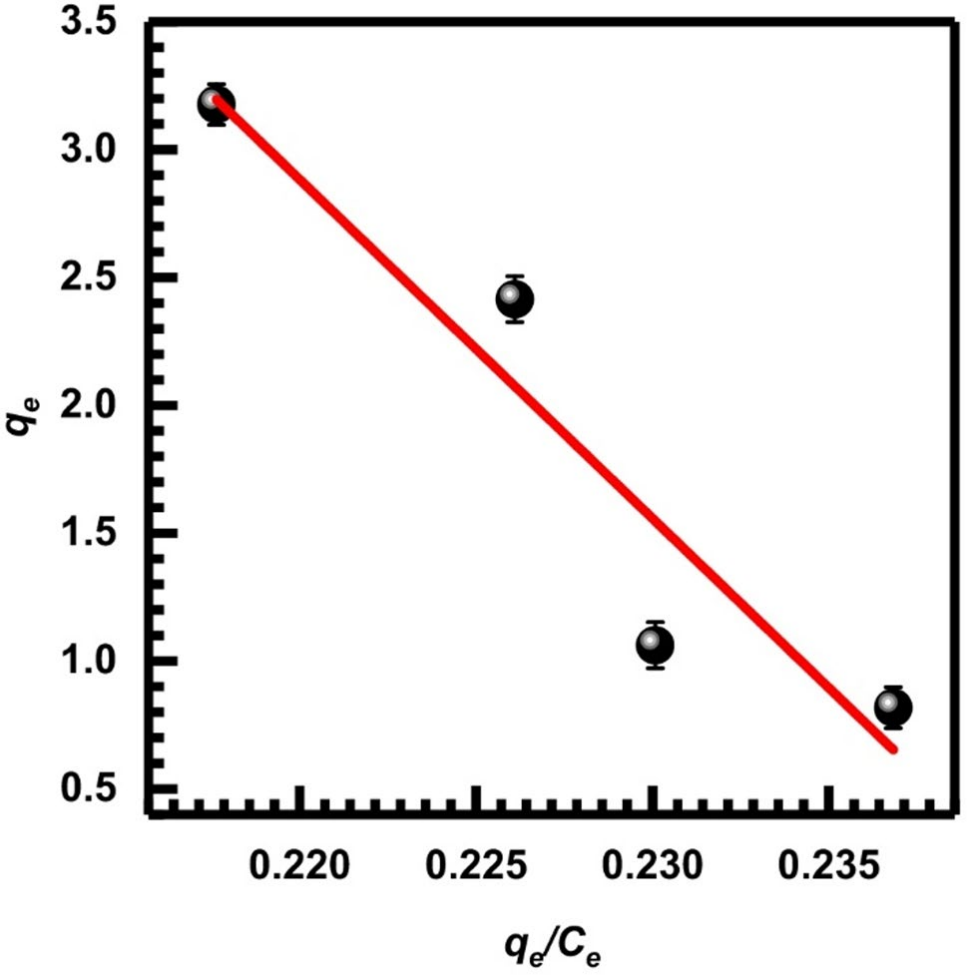
Isotherm of Langmuir-3, Eq. (11)

**Fig. 16:**
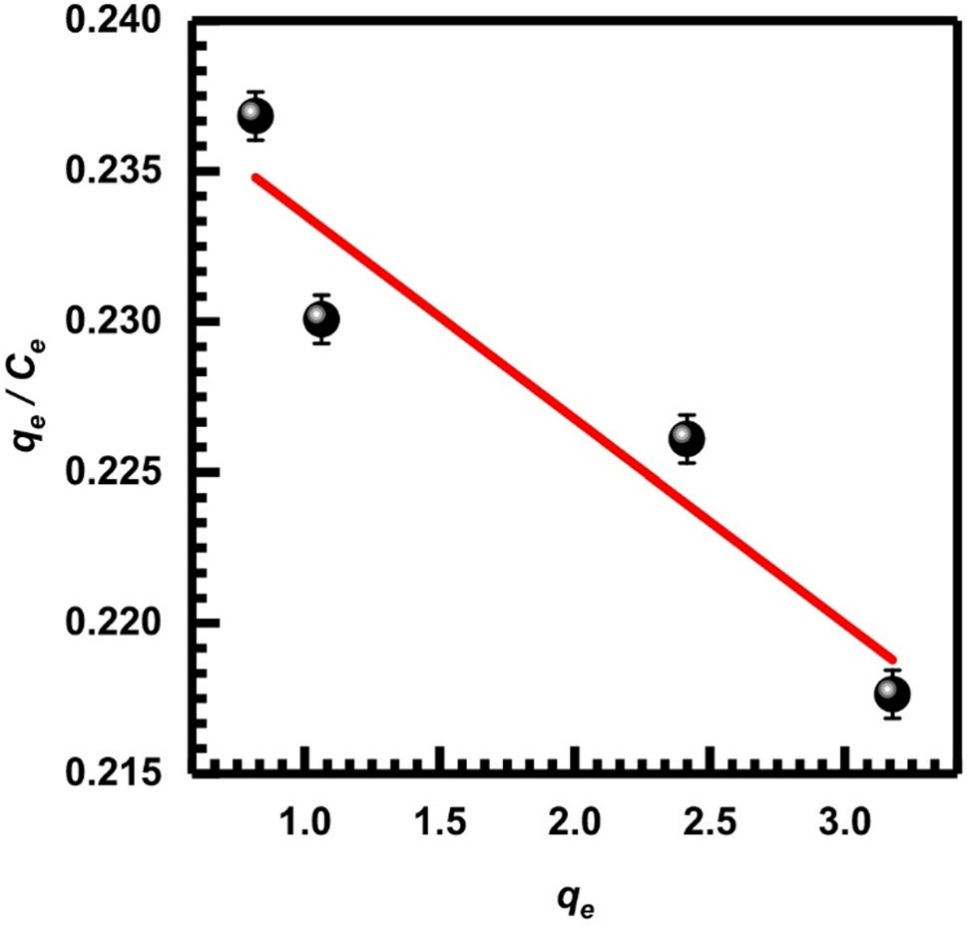
Isotherm of Langmuir-4, Eq. (12)

Here, *q*_*e*_ is the amount of metal ions retained per gram of adsorbent, whereas *C*_*e*_ (mg.L⁻¹) indicates the equilibrium concentration of the metal ion in the solution. The assumption of monolayer coverage is used to signify the adsorption capacity of the adsorbent surface at its theoretical maximum, *Q*_*m*_ (mg/g), and $\:{K}_{L}$ (LAA/mg), also known as the Langmuir constant, measures the affinity and binding strength of the adsorbent toward metal ions and describes adsorption behavior at low residual metal concentrations [71]. These values can be determined from the corresponding linearized plots as shown in Figs. 13, 14 and 15, and 16, in which experimental data of *C*_*e*_/*q*_*e*_ were plotted versus *C*_*e*_ (Langmuir-1); similarly, 1/*q*_*e*_ versus 1/*C*_*e*_ (Langmuir-2), *q*_*e*_ versus *q*_*e*_/*C*_*e*_ (Langmuir-3), and *q*_*e*_/*C*_*e*_ versus *q*_*e*_ (Langmuir-4). Values of *Q*_*m*_, $\:{K}_{L}$, and the correlation coefficient indicate the four versions of the Langmuir model. Table 2 displays the statistical results for the above models. According to the value of correlation coefficients of the four models, it is clear that the Langmuir-2 model is the best model to obtain *Q*_*m.*_ The *Q*_*m*_ of model 2 is 34.483, which suggests an important separation parameter that can give information on the type of isotherm, whether irreversible or favorable, given as a dimensionless parameter *R*_*L*_ [72].

13 $$ R_{L} = {\text{ 1}}/({\mathrm{1}} + b) $$

$\:{C}_{o}$ represents the initial metal ion concentration, while b represents the Langmuir constant. Given the value of *R*_*L*_, it may be determined if the isotherm is favorable (with *R*_*L*_ ranging from 0 to 1), linear (with *R*_*L*_ equal to 1), unfavorable (with *R*_*L*_ greater than 1), or irreversible (with *R*_*L*_ equal to 0). The calculated *R*_*L*_ according to model 2 being (0.4) which indicates favorable adsorption onto SL.

**Table 2:** Adsorption parameters of Cr (VI) onto SL at 25 °C according to the Langmuir isotherm model

Langmuir isotherm models	Slope	Intercept	Q_m_ (mg/g)	K_L_ (L/mg)	*R* ^2^
Langmuir-1 (Eq. 9)	0.0325	4.1055	31.25	0.779 × 10⁻²	0.982
Langmuir-2 (Eq. 10)	4.1274	0.0293	34.483	0.702 × 10⁻²	1
Langmuir-3 (Eq. 11)	132.49	32.031	30.94	0.79 × 10⁻²	0.983
Langmuir-4 (Eq. 12)	125.48	30.575	34.714	0.7 × 10⁻²	0.983

Another approach to understanding the adsorption isotherms is the Freundlich isotherm model. The Freundlich isotherm assumes the uptake of metal ions occurs on the heterogeneous surface by multilayer adsorption, and the amount of adsorbate adsorbed increases when the concentration is raised [66]. The non-linear form of the Freundlich isotherm model is given by Eq. (14).

14 $$\:{q}_{e}={K}_{F}{C}_{e}^{\frac{1}{n}}$$

A measure of the divergence from linearity is the Freundlich constant, which is related to the bonding energy and is represented as *K*_*F*_ ( (mg/g)(L/mg)^(1*/n*)^). Following is an explanation of how the n-value shows the level of nonlinearity in the relationship between solution concentration and adsorption: For adsorption to be linear, n must be less than 1, and it must be a chemical process. For adsorption to be physical, it must be greater than 1 [65].

Equation (15) provides the Freundlich isotherm model in the linear form:

15 $$\:\mathrm{log}{q}_{e}=\mathrm{log}{K}_{F}+\left(\frac{1}{n}\right)\mathrm{log}{C}_{e}$$

**Fig. 17:**
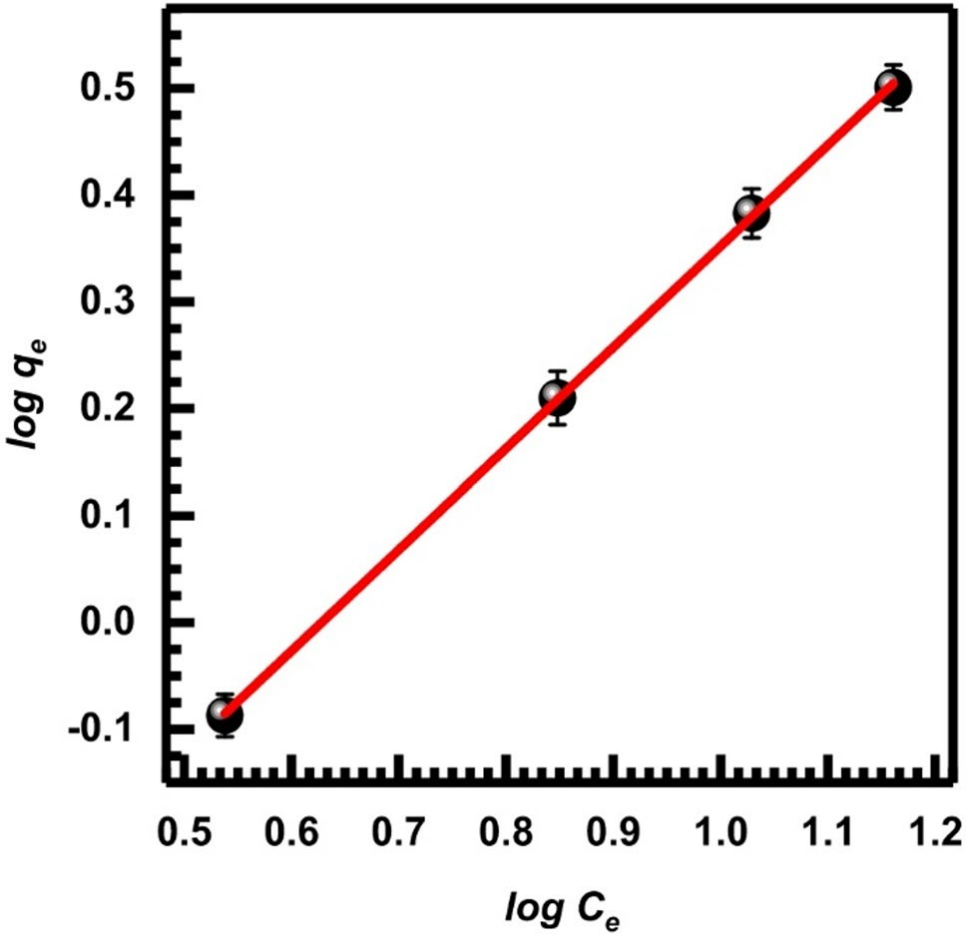
Freundlich isotherm, Eq. (14)

Figure 17 shows that for the given adsorption dose, the Freundlich equation was followed by the linear plots of log $\:{q}_{e}$ versus log $\:{C}_{e}$.Table 3 contains the relevant statistical data and Freundlich constants.

**Table 3:** The Freundlich and Temkin isotherm parameters and correlation values for Cr (VI) adsorption onto SL adsorbent at various doses

Isotherm model	Isotherm parameters	Isothermal values
Freundlich (Eq. 15)	Slope Intercept *K*_*F*_ n R^2^	0.947 0.595 2.440 0.666 0.992
Tempkin (Eq. 16)	Slope Intercept *A*_*T*_ *B*_*T*_ R^2^	1.537 1.195 14.174 3.415 0.924

The calculated *n* lies above zero and below one; hence, the process of adsorption may be considered as a physical process. However, the Langmuir-2 model gave a better correlation coefficient than the Freundlich model. It may be attributed to the fact that SL has a small surface area for Cr (VI) adsorption. Hence, only monolayer adsorption interaction takes place on the surface of SL [59].

The Tempkin isotherm model also accounts for the adsorbent-adsorbate interactions. Two key assumptions underpin the model: first, that the adsorbate is distributed uniformly, and second, that adsorbate-adsorbate repulsion causes the heat of adsorption to decrease linearly with the coverage of molecules in the layer. Another assumption is that the Freundlich isotherm does not hold and that the adsorption heat is decreasing linearly. The non-linear form of the Tempkin model is given by Eq. (16).

16 $$\:{q}_{e}=\frac{RT}{{b}_{T}}\mathrm{ln}\left({A}_{T}{C}_{e}\right)$$

The following is the linear version of the given equation:

17 $${q_e}={B_T}{\text{ln }}\left( {{A_T}} \right){\text{ }}+{B_T}{\text{ln }}\left( {{C_e}} \right)$$

**Fig. 18:**
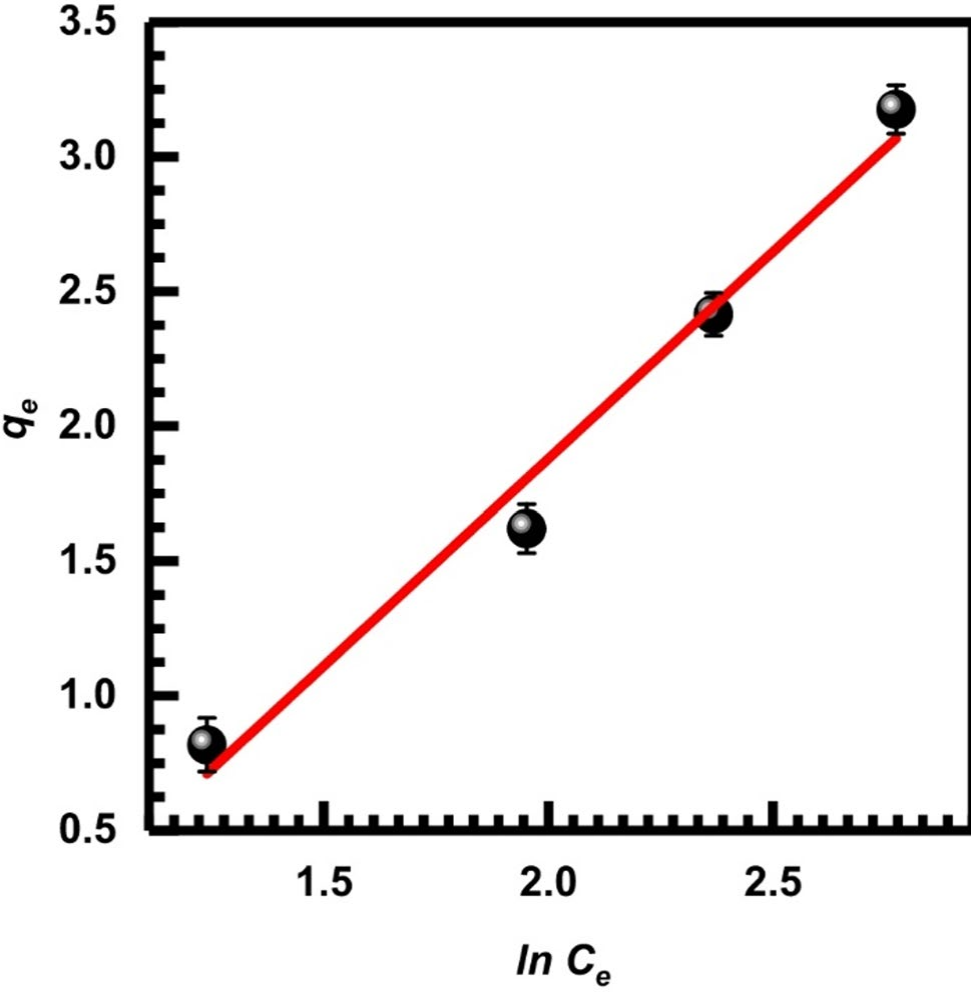
Tempkin isotherm, Eq. (17)

In this equation, AT is the equilibrium binding constant (L/mg) that corresponds to the largest binding energy, and BT is the heat of adsorption. Table 3 displays the values of the Tempkin constants, and Fig. 18 plots *q*_*e*_ against ln *C*_*e*_ at the investigated adsorbent doses.

The highest correlation coefficient (R²) was given by Langmuir-2 models of adsorption data when compared with others. A monolayer of Cr(VI) ions on the surface of the SL adsorbent was suggested by the Langmuir model, which provided a better fit than other adsorption kinetics models. The Tempkin model gave the worst correlation coefficient (Table 3). The failure of the Tempkin model can be attributed to the linear assumption of the decline of the heat of adsorption as a function of temperature rather than logarithmic as in the Langmuir and Freundlich models. This result agrees with previous investigations in which the Tempkin model failed to describe biosorption of several metals in Table 1, such as Ni (II) [73] Cu (II) [74] Cr (VI) [75]. It has been suggested [75] that the Tempkin isotherm model is incapable of modelling the bio-adsorption equilibrium, probably due to the original derivation assumption that the association is predicting gas phase equilibrium.

### Mechanism explanation (for Cr (VI) removal by pine cone adsorbent)

Pine-cone biomass surface functional groups (-OH, -COOH) become protonated and produce -OH₂⁺ groups at low pH (1–3). Electrostatic interaction causes these positively charged sites to attract Cr(VI) species (HCrO₄⁻, Cr₂O₇²⁻) that are negatively charged. Because of the presence of reducing agents based on phenolic and lignin-based compounds, Cr(VI) is partially reduced to Cr(III). This process begins with adsorption, continues with reduction, and culminates in complexation, where stable surface-metal complexes are formed by chelating or complexing the reduced Cr(III) on the surface. According to this kinetic model, chemical interactions are formed between the adsorbate and the surface-located adsorbent functional groups. This is supported by the low surface area measured by BET, which suggests that the quantity of mesopores and micropores was extremely low. The comparison studies pertaining to the usage of pine cone with different conditions as an adsorbent for the removal of heavy metals from wastewater are displayed in Table 4.

**Table 4:** Comparison of studies using pine cones-based adsorbents (sorted by year)

No.	Reference (year)	Type of material	Target metal (s)	Activation/modification	Best conditions/results	Isotherm model	Kinetic model	Mechanism
1	Momčilović et al. (2011) [76]	Activated carbon from Pinus nigra cones	Pb(II)	Chemical activation (H₃PO₄)	Max adsorption ≈ 98 mg/g at pH 5	Langmuir	Pseudo-second order	–
2	Saif (2015) [77]	Activated carbon from Pinus roxburghii cones	Cu(II), Ni(II), Cr(III)	Thermal + acid activation	Cu(II): 92% removal at pH 6	Freundlich	Pseudo-second order	–
3	Ben Amar (2021) [78]	Raw pine cone powder	Cd(II), Pb(II)	None (raw biosorbent)	75–80% removal at pH 6	Langmuir	Pseudo-second order	–
4	Ben Amar et al. (2024) [79]	Pine cone shell (raw & treated)	Pb(II), Cu(II), Cd(II), Ni(II), Cr(VI)	NaOH-treated & dried	Pb(II): 90% removal; Cr(VI): 84% at pH 2	Langmuir	Thomas & Yoon–Nelson	Electrostatic attraction + reduction of Cr(VI) → Cr(III) + surface complexation
5	Chyad (2024) [80]	Pine cone activated carbon (PCAC)	Cu(II)	HCl activation	89% Cu(II) removal at pH 5.5, 60 min	Freundlich	Pseudo-second order	–
6	Macena et al. (2025) [81]	Raw pine cone powder	Zn(II)	None (raw biosorbent)	~ 96% removal at pH 7; max capacity = 7.92 mg/g	Freundlich	Pseudo-second order	Chemisorption via –OH/–COOH functional groups

## Conclusion

Sulfonated lignin (SL) can be a sustainable and cost-effective alternative to conventional adsorbents for removing hexavalent chromium (Cr⁶⁺) from contaminated water, as demonstrated in this study. Adsorption performance was highly dependent on pH, with maximal Cr⁶⁺ uptake at pH 2 and 10 mg.L⁻¹ starting metal ion concentration. The Langmuir isotherm model best explains equilibrium adsorption data, revealing monolayer adsorption with a maximal capacity of 34.483 mg.g⁻¹. The Freundlich and Temkin models also correlated well, confirming heterogeneous and multilayer SL surface interactions. Kinetic experiments confirmed chemisorption as the primary mechanism for adsorbing Cr⁶⁺ ions, following a pseudo-second-order model.SL had comparable or superior absorption capacity and efficiency than lignin-based or agricultural biosorbents, demonstrating sulfonation’s ability to enhance surface functional groups and active sites. This breakthrough advances green and high-performance heavy metal biosorbents. In summary, the prevalence of functional groups including hydroxyl, carboxyl, methoxy, and phenolic groups in SL’s chemical makeup underscores their potential as efficient biosorbents. These groups, which are mostly found in cellulose, hemicelluloses, and lignin, contribute to a variety of metal ion interactions, such as complexation, ion exchange, coordination, and electrostatic attraction. Future study should assess its reusability and regeneration capacity to improve its environmental and economic worth and enable the creation of sustainable adsorption and catalytic systems for large-scale water purification.

## Data Availability

All data generated or analyzed during this study are included in this published article.

## Abbreviations

SEM: Scanning electron microscopy
FTIR: Fourier transform-infrared spectrum
BET: The Brunauer–Emmett–Teller
SL: Sulfonated lignin
mg L^− 1^: Milligrams per liter
